# Speech as an indicator for psychosocial stress: A network analytic approach

**DOI:** 10.3758/s13428-021-01670-x

**Published:** 2021-08-06

**Authors:** Mitchel Kappen, Kristof Hoorelbeke, Nilesh Madhu, Kris Demuynck, Marie-Anne Vanderhasselt

**Affiliations:** 1 Department of Head and Skin, Department of Psychiatry and Medical Psychology, , Ghent University, University Hospital Ghent (UZ Ghent), Corneel Heymanslaan 10-13K12, 9000 Ghent, Belgium; 2 Ghent Experimental Psychiatry (GHEP) Lab, Ghent University, Ghent, Belgium; 3 Department of Experimental Clinical and Health Psychology, Ghent University, Ghent, Belgium; 4 IDLab, Ghent University – imec, Ghent, Belgium

**Keywords:** Voice stress analysis, Speech, Stress, Psychological stress, Sympathetic nervous system, Fundamental frequency, Jitter, Harmonics-to-noise ratio, Voiced speech

## Abstract

Recently, the possibilities of detecting psychosocial stress from speech have been discussed. Yet, there are mixed effects and a current lack of clarity in relations and directions for parameters derived from stressed speech. The aim of the current study is – in a controlled psychosocial stress induction experiment – to apply network modeling to (1) look into the unique associations between specific speech parameters, comparing speech networks containing fundamental frequency (F0), jitter, mean voiced segment length, and Harmonics-to-Noise Ratio (HNR) pre- and post-stress induction, and (2) examine how changes pre- versus post-stress induction (i.e., change network) in each of the parameters are related to changes in self-reported negative affect. Results show that the network of speech parameters is similar after versus before the stress induction, with a central role of HNR, which shows that the complex interplay and unique associations between each of the used speech parameters is not impacted by psychosocial stress (aim 1). Moreover, we found a change network (consisting of pre-post stress difference values) with changes in jitter being positively related to changes in self-reported negative affect (aim 2). These findings illustrate – for the first time in a well-controlled but ecologically valid setting – the complex relations between different speech parameters in the context of psychosocial stress. Longitudinal and experimental studies are required to further investigate these relationships and to test whether the identified paths in the networks are indicative of causal relationships.

## Introduction

Stress is an increasingly relevant topic in modern society, with the majority of people experiencing regular stress symptoms. Considering the broad range of physiological and psychological factors that are influenced by stress, a plethora of methods have been developed to assess individuals’ stress levels. Currently, commonly used methods determine stress levels by self-report questionnaires on factors that are affected by stress (e.g., mood) or broader indicators of psychological well-being (Monroe, 2008). Besides self-reports, stress is also commonly assessed through the measurement of biological processes involved with stress exposure. The main advantage of measuring stress through biomarkers, as compared to interviews and self-report instruments, is that these measures are not subject to self-report biases. Furthermore, biomarkers allow continuous monitoring of stress levels. Many different biological markers of stress have been identified such as heart rate, blood pressure, cortisol, skin conductance, and many more (for an extensive overview, see: Fink, 2017; Shields & Slavich, 2017). Even though many of these methods are highly effective in determining one’s stress levels, they are often costly, requiring the attachment of electrodes (e.g., electrocardiography; ECG) or the extraction of a blood or saliva sample, and demanding since they generally require interaction with a physician or expert and specialized apparatus to collect the data. With the emerging market of wearables (e.g., smartwatches), it has become increasingly easy to collect continuous data of stress-related physiological markers such as heart rate, skin conductance, and skin temperature. Although the quality of these methods is constantly improving, it is not always evident to continuously collect this data (e.g., costs, privacy) besides often reported problems with regards to continuity of its accuracy (e.g., loss of connection). Therefore, the need to further explore alternatives for stress measurements remains. Recently, speech analysis has been proposed as a possible physiological marker for stress, however, further research is required (Giddens et al., 2013; Slavich et al., 2019).

Speech production is a complex process that requires the involvement of many different parts of the body. To produce speech, one first considers what words to say, tone of voice, and many more conscious aspects. However, the practical part happens more automatically, which is the actual sound production. When producing speech, the body modulates the tension of numerous muscles to push air through the vocal folds and out the vocal tract to produce sound waves (Titze & Martin, 1998). Since stress increases both muscle tension and respiration rate, which in turn influence speech production, it has been proposed that stress should be detectable from the way speech sounds (Sondhi et al., 2015). A major advantage of stress detection from speech is the non-intrusive obtainability of speech data and the possibility of swift, cost-effective, and remote stress assessments. As such, speech is considered a promising psychophysiological measure for stress assessment.

However, relatively little is known with regards to how specific speech parameters interact in a context with or without stress, and how this interaction of speech parameters changes following a stressor. That is, speech research in the context of stress is still in its infancy and has mostly flourished at the fundamental level of parameter identification and development. Speech consists of many different parameters (i.e., characteristics), which are contingent on many factors, both conscious and automatic. Fundamental frequency, Harmonics-to-Noise Ratio, and jitter are such speech parameters that have been found to change in stressed subjects (Giddens et al., 2013; Kreiman & Sidtis, 2011; Mendoza & Carballo, 1998; Orlikoff, 1990; Orlikoff & Baken, 1989). Based on the available literature, *1) Fundamental frequency (F0)* can be considered a key speech parameter in the context of different types of stressors. F0 refers to the frequency at which the vocal cords vibrate, and gives rise to the idea of the *pitch* of the voice. Research suggests a universal trend of increase in F0 in stressed subjects (Giddens et al., 2010, 2013; Godin & Hansen, 2008; Johannes et al., 2007; Koblick, 2004; Kreiman & Sidtis, 2011; Mendoza & Carballo, 1998; Rothkrantz et al., 2004; Williams & Stevens, 1972). Another widely used speech parameter is *2) Harmonics-to-Noise Ratio (HNR),* which indicates one’s vocal quality by measuring the additive noise in the speech signal during voiced periods (e.g., when uttering vowels). HNR has mainly been studied in physical stress tasks (e.g., workout), and has shown to decrease with increased physical task stress (Godin et al., 2012; Godin & Hansen, 2015; Koblick, 2004), but has shown mixed results in the context of cognitive load/psychological stress (e.g., tongue twister, reciting the alphabet backwards; Mendoza & Carballo, 1998). *3) Jitter* refers to the frequency variation from cycle to cycle and has been found to reduce in the context of stress, however this trend has not shown to be universal (Giddens et al., 2013; Mendoza & Carballo, 1998). Moreover, *4) formants* have been opted as promising features of speech in distinguishing stress from speech, more specifically, the shifting of formant 1 (F1) and formant 2 (F2) have shown to be decent indicators of psychological stress (Sigmund, 2012; Van Puyvelde et al., 2018). Formants are the primary resonances of the vocal tract and can shift due to numerous conscious and unconscious processes and are dependent on one's speech style (Shahin & Botros, 2001). There has, however, not been consensus on the effects of psychological stress on F1 and F2, which indicates it to be heavily influenced by individual trends rather than global trends valid for all speakers (Kirchhuebel, 2010; Sigmund, 2012). Since both change in F1 and F2 play a role in stress, a ratio score could be computed that is reactive to changes in either formant; formant 1:2 ratio. Lastly, it has been suggested that with increased physical stress, breathing patterns and muscle tension impact different aspects of speech, such as inappropriate pause placements (Van Puyvelde et al., 2018). As a final feature, *5) Mean voiced segment length* can be used to gain insight into such speaking patterns, as it is the mean length of the continuously voiced regions which is expected to decrease under stress.

Even though research is currently lacking, the combination of these speech parameters is highly promising for the detection and understanding of increased stress. However, it should be noted that each of these parameters reflect unique features of a complex speech production process. Therefore, these parameters are highly interrelated, where the unique interplay between each of these measures remains to be modeled. In particular, little is known regarding the complex interplay between speech parameters and how it is affected by stress (Giddens et al., 2013; Kreiman & Sidtis, 2011). Much of the recent work in stress detection from speech has been conducted in controlled, quiet lab settings or with vocal actors acting out a stressful monologue rather than truly experiencing psychological stress (Giddens et al., 2013), limiting the ecological validity of previous findings. Moreover, it has been suggested that the effect of stress on the formants, which are shifted as a consequence, and jitter is heavily influenced by individual differences in stress reactivity (Giddens et al., 2013; Scherer, 1986). This is likely to also be the case for other speech parameters and could explain the mixed results observed in the literature. Considering the variety of environments, microphones with different qualities, and interindividual differences in stress expression in speech, a number of researchers have expressed the need for high-quality studies, using real participants rather than voice actors, to compose large datasets of speech data with high-quality stress labels and recorded in a variety of contexts (Giddens et al., 2013; Slavich et al., 2019). Moreover, many researchers investigated stressful versus non-stressful events in their experimental designs without verifying whether participants truly experienced stress by using physiological markers or inquiries. Lastly, previous research has primarily focused on how the entirety of indicators is associated with stress, without highlighting the complex dynamics amongst the indicators and how each of the speech features are uniquely related to stress.

The current study uses a design that takes the above-mentioned shortcomings into consideration to establish a common ground from which new insights can be developed. Healthy individuals will be instructed to read out loud standardized texts both prior to and after exposure to a highly controlled psychosocial stressor. Psychosocial stressors are often described as one of the most powerful and ecologically valid stressors (Kirschbaum & Hellhammer, 1994). Psychosocial stress is induced in situations of social evaluation, social exclusion, and other situations in which social threat occurs (Dickerson & Kemeny, 2004). The need to be associated with others and to maintain a social-self are core psychological needs (Panksepp, 2003; Tossani, 2013). When one of these needs is threatened, for example when being negatively compared to others, social threat and thus stress is induced (Dickerson & Kemeny, 2004). Social evaluation induces an increased stress response, which is expressed in increased electrodermal activity (i.e., skin conductance), subjective (experienced) stress, and negative affect (Dedovic et al., 2009; Dickerson & Kemeny, 2004).

Given that our literature review demonstrates mixed effects for parameters derived from stressed speech (and thus a lack of clarity in their relations and direction), and that the interrelation between each of these constructs in the context of stress (i.e., speech parameters, skin conductance levels, and self-reported mood) remains to be explored, we will make use of psychological network models (Borsboom & Cramer, 2013; Newman, 2010). Network methodology is an increasingly used technique to gain insight into complex relationships in a data-driven manner, allowing mapping how each of the constructs of interest is uniquely related to one another. As such, network models are well suited to explore whether and how the complex interplay between each of the above-presented core speech parameters is impacted by stress. In addition, network analysis allows us to map how changes in speech due to experimental manipulation of stress relate to changes in negative affect. This study has two main aims: 1) We aim to model the impact of a psychosocial stressor (the Montreal Imaging Stress Task (MIST); Dedovic et al., 2005) on the unique associations between the speech parameters of interest (fundamental frequency, jitter, Harmonics-to-Noise Ratio, formant 1:2 ratio, and mean voiced segment length) before and after the stressor (aim 1); 2) we will model how stress induced change in the speech parameters relates to change in the negative affect ratings (measured with VAS) as these analyses will shed light on the unique associations between the change in speech features and negative affect following a psychosocial stressor (aim 2). Given the exploratory data-driven approach and undirected nature of the models, the obtained network models are likely to allow further hypothesis generation, which will be informative for future confirmatory studies.

## Methods

### Participants

A convenience sample of 148 students (M age = 26.7, SD age = 12.5, 51 female, 97 male) was recruited through flyers, social media, and University of Ghent mailing lists informing them on the duration of the experiment, the possibility to win a 25 euro gift card, and a link to www.vopexperiment.be where participants could plan their session through a youcanbookme synchronization. The study was conducted in accordance with the ethical guidelines of the Faculty of Psychology and Educational Sciences of Ghent University, and all participants gave written consent before participating.

#### Apparatus and procedure

Participants were seated in one of two nearly identical rooms in front of a Huawei MediaPad M5 tablet. The task was written in Java using Android Studio. Before any instructions commenced, participants signed the informed consent form. Then, participants were instructed on the procedure, how the tablet and application worked (how to record, etc.), and the cover story (cf. infra) was repeated to minimize the likelihood of the actual purpose of the study being identified. Next, participants were given a smartwatch (Chill+ Band) to put on their dominant hand (from which electrodermal activity was measured; EDA), and ECG electrodes were placed on the sternum. The participants were informed on the purpose of each of these measures with the cover story of it being used to validate the smartwatch measures. Data quality was shortly inspected before the actual experiment started. Firstly, participants were requested to rate the VAS. Next, participants were instructed to rest for 5 min to ensure they were relaxed and to minimize the impact of any events occurring previous to the experiments (e.g., rushing or nervousness). Following the resting phase, participants were instructed to read-out-loud a five-sentence piece of text that was the same for all participants and an often-used text in Dutch speech therapy:

*“Papa en Marloes staan op het station. Ze wachten op de trein. Eerst hebben ze een kaartje gekocht. Er stond een hele lange rij, dus dat duurde wel even. Nu wachten ze tot de trein eraan komt. Het is al vijf over drie, dus het duurt nog vier minuten. Er staan nog veel meer mensen te wachten. Marloes kijkt naar links, in de verte ziet ze de trein al aankomen.”* From: van de Weijer and Slis (1991)

Participants were instructed that the recording of the speech was to train speech-to-text algorithms to hide the actual purpose but to ensure the accurate pronunciation of the text. Next, once again, the VAS sliding scales were answered, providing a baseline measure for NA in a relatively unstressed state (i.e., following the first resting block). After that, the MIST (see Stress induction procedure header) commenced, starting with instructions and 2 min of practice trials during which no social comparison was made and without a trial time limit. After the testing phase of the MIST, another speech recording and VAS segment was conducted, which corresponds to the post-stress measurement. The end phase of the experiment consisted of another 5-min resting block to prevent participants from leaving the experiment in a stressed state, followed by another block of VAS questions. The experiment was concluded by conducting the Ruminative Response Scale (RRS) and the Depression, Anxiety, and Stress Scale (DASS) in order to get an estimation of the sample characteristics. At the end, the participants were debriefed and informed on the actual purpose of the study.

#### Stress induction procedure

In order to induce acute stress in our participants and investigate the effects of stress on networks of speech parameters, we used the Montreal Imaging Stress Task (MIST; Dedovic et al., 2005). This is a sequence of arithmetic questions designed as a stress induction task. To ensure a proper understanding of the task, participants could practice for 2 min. Trials consisted of mathematical tasks where the correct answer was situated between 0 and 9. Participants were instructed to answer these trials as quickly as possible using arrow buttons to select the right answer on a number wheel. After the practice block, the actual task started and was performed for 5 min. During this task, participants were shown a time limit per trial, which was set at 90% of their average time during the practice block. In addition, time limits were reduced by another 10% when they answered three consecutive trials correctly. Throughout the task, participants were presented a performance indicator showing their performance as compared to the *‘average participant’*, which in reality was an unfeasible, fictional benchmark. Participants were instructed that they should not deviate from the average performance too much, and that if they would the data would be unusable for the purpose of the study. Throughout the task, the experimenter was sitting across from the participant and taking notes. Since the participant is always performing worse than the *‘average participant’*, and with the experimenter taking notes and the constant time pressure, stress is induced. As a cover story, participants were told that the study attempts to link biometric signals to quick arithmetic solving skills.

#### Trial

Trials showed a numbered wheel from 0 to 9 on which the participant could select the desired answer using arrow buttons and confirm when ready. Above the numbered wheel, an arithmetic task was presented. The top of the screen showed a red bar that was slowly disappearing, indicating the time left for that specific trial. Another bar was shown representing how well the participant was performing compared to others, which was always negative. After answering, participants were either shown a green overlay saying *correct* or a red overlay saying *incorrect*. If they ran out of time, a red overlay saying *timeout* was presented. The practice trials, which were offered at the beginning of the task, did not have a time limit and did not show a comparison to other participants. These were used to familiarize the participant with the task as well as getting a reference reaction time to calculate the trial time limits in the experimental phase. See supplemental material for screenshots.

#### Self-report measurement – negative affect; NA

To evaluate negative affect (NA) as an indicator of stress, self-reported mood was measured at four time points (baseline [T1], pre-stress [T2], post-stress [T3], post-recovery [T4]), by using the three NA items of a seven-item mood questionnaire (adopted from the Profile of Mood States (POMS); Rossi & Pourtois, 2012) presented on a sliding scale from 0 to 100 on different states (angry, tense, dejected). The answers to these VAS are used as a manipulation check for the stress induction procedure. More specifically, we used the items representing negative affect (angry, tense, dejected) allowing a compound score for NA (ranging from 0 to 100) where high scores reflect being in a more negative mood state.

#### Extraction of speech parameters

Speech parameters were extracted using OpenSmile 2.3.0 (Eyben et al., 2010) and the GeMAPS configuration (Eyben et al., 2015), a parameter set used in voice research and affective computing. Fundamental frequency is the central tendency of the frequency of vibration of the vocal folds during speech, and as such, is closely related to pitch, which is defined as our perception of fundamental frequency. Jitter is the deviation in the F0 computed across consecutive time segments. Formant 1:2 ratio is the ratio of the energy of the first formant (F1) to the energy of the second formant (F2). Harmonics-to-Noise Ratio (HNR) is the relation of energy in harmonic components to energy in noise-like components, and lastly, mean voiced segment length is the average length of continuously voiced regions (F0 > 0), thus sounds made while the vocal cords vibrate. For more detailed information on parameter calculation and extraction procedure, we refer the reader to Eyben et al. (2010) and Eyben et al. (2015) and the Supplemental Material.

#### Statistical analyses

The network analyses were conducted in R (for detailed version information of the statistical software and packages used, see supplemental materials). As part of the manipulation check, we fitted generalized linear mixed models (GLMMs) using the ‘lme4’ (Bates et al., 2014) and ‘car’ (Bates et al., 2014; Fox et al., 2012) packages. The sum of squares for the models was estimated using the type III approach, and the statistical significance level was set to *p* < .05. Follow-up tests with pairwise comparisons of the estimated marginal means (EMMs) were performed with the ‘emmeans’ R Package (Lenth, 2018).

We relied on Gaussian graphical models (GGMs), also referred to as regularized partial correlation networks, to model the impact of stress on the unique associations between the speech parameters of interest (fundamental frequency, jitter, Harmonics-to-Noise Ratio, formant 1:2 ratio, and mean voiced segment length), as well as the relation between change in speech parameters and change in NA throughout the stress induction procedure. For this purpose, we estimated three separate GGMs. In particular, we computed: (1) a network including each of the speech parameters of interest, assessed following a resting phase (referred to as *resting state network*), (2) a network including the speech parameters, assessed immediately following the stress induction procedure (referred to as *stress network*), and (3) a network including the change scores for each of the speech parameters and the compound measure for NA (referred to as *stress reactivity network*). Change in NA / speech parameters was computed by subtracting the resting state measure from the post-stressor measure. As such, a positive value reflects an increase in NA / the speech parameters throughout the induction procedure.

##### Data preparation and network estimation

To improve normality, all variables underwent nonparanormal transformation using the *huge* package (Zhao et al., 2012), after which the GGMs were estimated using the *qgraph* package (Epskamp et al., 2012). As the name suggests, GGMs or regularized partial correlation networks depict the unique associations (partial correlations) between each of the variables (referred to as “nodes”) included in the analyses. In network models, the unique associations between each of the nodes are referred to as “edges“. However, given that absence of an association between two constructs does not always result in a correlation coefficient of exactly zero, the need arises for a phase of regularization to prevent the inclusion of spurious associations. For this purpose, we relied on the Graphical Least Absolute Shrinkage and Selection Operator (gLASSO; Friedman et al., 2014), which shrinks small associations, likely reflecting spurious / false-positive findings, to zero (similar to multiple comparison corrections, for more information see Friedman et al., 2014 and Epskamp & Fried, 2018 for a tutorial on GGMs including this regularization technique). The model with the best fit was then selected using the extended Bayesian information criterion with hyperparameter γ = 0.5. This hyperparameter setting errs on the side of parsimony, maximizing model specificity (Epskamp & Fried, 2018). As a result, the obtained network model is less likely to include false-positive associations (for a more detailed discussion of estimation of GGMs, including an extensive tutorial, see Epskamp & Fried, 2018). To examine which nodes take a more central role in the model, we estimated node strength centrality. Strength centrality is calculated as the sum of absolute edge weights connected to each node in the model (Costantini et al., 2015). As such, high scores on strength centrality reflect that the node is more strongly connected. Finally, we used a node-wise regression approach to estimate node predictability, the proportion of variance of each node that is explained by its neighboring nodes (Haslbeck & Fried, 2017). For this purpose, we relied on the *mgm* package (Haslbeck & Waldorp, 2015).

##### Network visualization

The network models were plotted with *qgraph*, using a modification of the Fruchterman–Reingold’s algorithm (Fruchterman & Reingold, 1991). This algorithm aims to position nodes in the network based on their level of connectivity (but see Jones et al., 2018). Unique associations between nodes are represented by edges. The thickness of each of the edges reflects the strength of the association, whereas the color and type of line (full/dashed) reflects the valence of the edge (blue/full: positive association; red/dashed: negative association). The GGMs are undirected and as such allow no interpretation regarding the direction of effects. To facilitate visual comparison between the resting state and stress network, the layout of these two networks was constrained to be identical (using the average layout of both models). In addition, for these two networks, we plotted the thickness of each of the edges relative to the strongest edge observed over both models. Moreover, for each of the nodes, we present the proportion of explained variance by the neighboring nodes as a pie chart in the outer ring of the node (node predictability). Strength centrality was standardized to facilitate interpretation.

##### Network comparison

To compare the resting state network and stress network, we first correlated the two obtained adjacency matrices. Similarly, we examined how the estimates of Strength centrality obtained for each of the network models correlated, as well as node predictability. We then proceeded with permutation tests for network structure invariance, allowing to test whether the network structures significantly differed, and global strength invariance, testing potential differences between the resting state- and stress network in (overall) strength of connectivity (van Borkulo et al., 2017). For this purpose, we relied on the *NetworkComparisonTest* package (for dependent samples; van Borkulo et al., 2016).

##### Evaluation of the stability and accuracy of the models

To evaluate the stability and accuracy of each of the obtained network models, we followed bootstrapping procedures set-out by Epskamp, Borsboom, and Fried (2018). In particular, using the *bootnet* package (Epskamp & Fried, 2017) we modeled sampling variability in edge weights (edge accuracy) and plotted significant differences in edge weights. Furthermore, we evaluated the stability of the indicator of node centrality, modeling the extent to which the order of strength centrality remained stable in subsets of the data (cf. case-dropping subset bootstrap). To be considered stable, the corresponding correlation stability coefficient should be ≥ .25 (Epskamp et al., 2018).

## Results

Due to technical malfunctions, all ECG data was unusable and a part of the EDA has not been collected properly for some participants throughout the experiment (*n* = 32). Therefore, the EDA data (together with self-reported mood data) that was collected accurately is used to validate the stress induction method, but will not be included in the network analyses (*n* = 148).

## Manipulation check

Before the main analysis, a manipulation check was conducted to verify whether the stress induction was successful by comparing both negative affect (NA) pre- and post-stress induction, and EDA pre-, during-, and post-stress induction. Given the non-normality of the EDA data, a series of (G)LMM (generalized linear mixed models) were conducted to ensure the use of a statistical model that best fits the underlying distribution (e.g., normal, gamma). Based on the Akaike information criterion (AIC), EDA was best described by a gamma model with a log-link (AIC = 802.5).

**Fig. 1:**
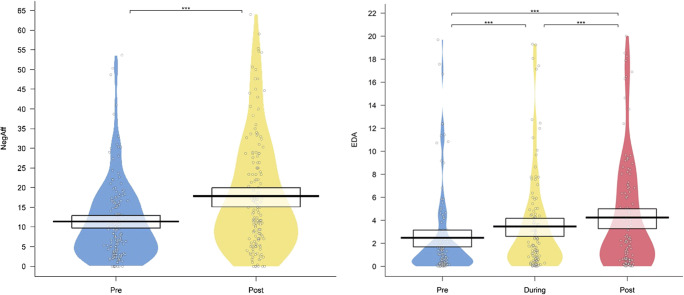
a; *left* Negative affect pre- and post-stress induction. b; *right* EDA pre-, during, and post-stress induction

Corresponding models were fit with only *time* (pre - post for negative affect (two levels) and pre - during - post for EDA (three levels)) as an independent variable and subject ID as random intercept. The LMM for negative affect showed a significant effect of time (see Fig. 1a), *p <* .001 with post-stress scores showing significantly more negative affect than pre-stress, *b* = 6.45, SE = .877, *t* = – 7.35, *p <* .001. Moreover, the GLMM for EDA also showed a significant effect of time (see Fig. 1b), *χ*^*2*^ = 247.59*, p <* .001, with EDA increasing during the task versus prior to the task, *b* = .621, SE = .029, *z* = – 10.29, *p <* .001, EDA after the task being higher than during the task *b* = .776*,* SE = .035, *z* = –5.53, *p <* .001 and EDA after the task being higher than prior to the task, *b* = .482, SE = .023, *z* = –15.513, *p <* .001.

To further underline the stress induction effectiveness, we ran a Pearson correlation between the delta scores computed for EDA and negative affect. The delta scores were computed by subtracting the data from the resting state measure from the post-stressor measure for these variables, resulting in scores that indicate an increase after the stress induction when positive, and a decrease when negative. A significant correlation, *r*(114) = .19, *p* = .04, was found following the expected trend of negative interrelatedness, therefore supporting the stress-induction method.

## Impact of stress on the interrelations between speech parameters (aim 1)

**Fig. 2:**
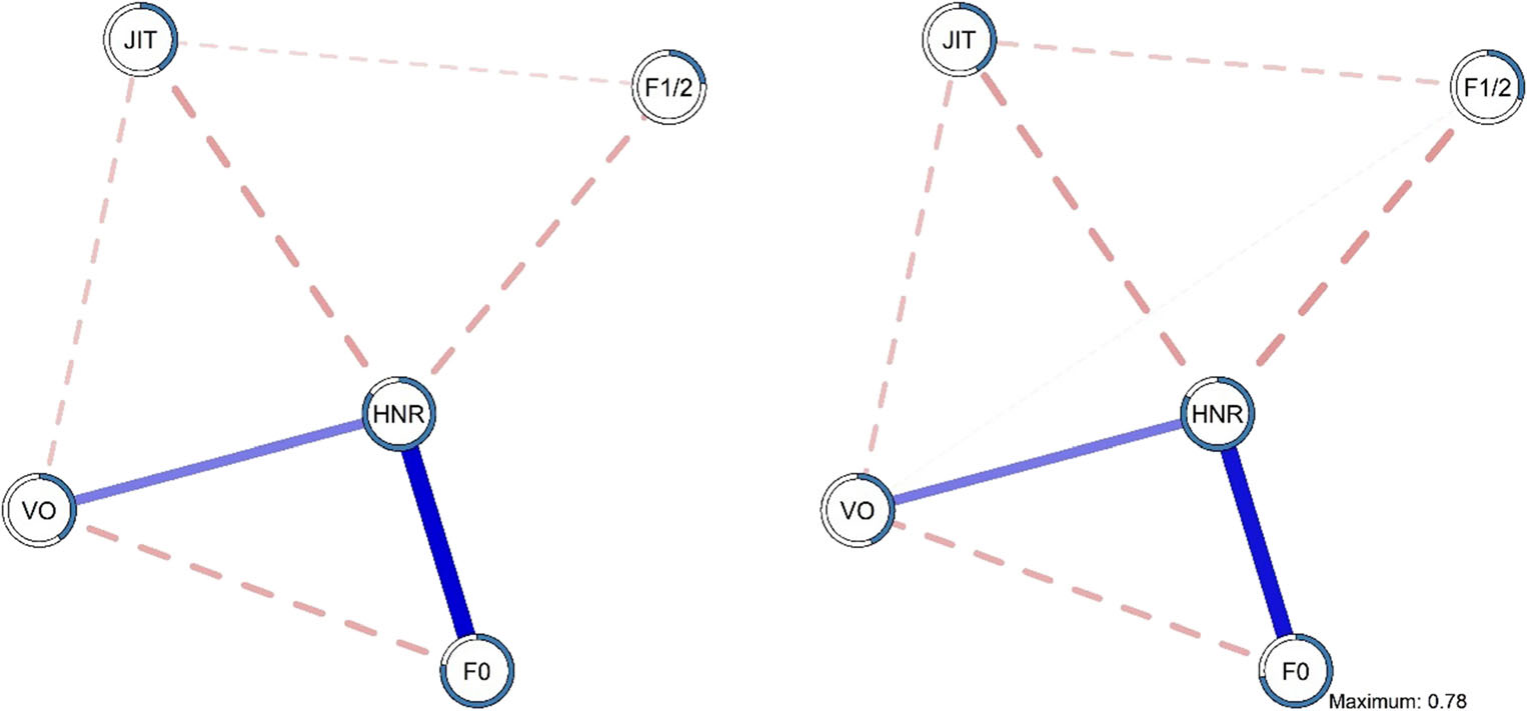
Unique associations between the voice parameters pre- (a; *left*) and post-stressor (b; *right*). *Note.* Edges in the models represent the unique associations between each of the nodes. Edge thickness reflects the strength of association, where strong associations are presented using thicker edges. *Blue/Full edges* represent positive associations, whereas *red/dashed edges* represent negative associations; the edge weights presented in the model can also be found in the edge weight matrix (Supplemental Tables 6, 7). Node predictability (R^2^) is visualized as a pie chart around each node and can also be found in Supplementary Table 1

Our first aim was to model the impact of stress on the interrelations between the speech parameters of interest by comparing a pre-stressor network with a post-stressor network. These networks consist of nodes representing variables, connected by edges representing regularized partial correlations. As such, every edge (connection) between two nodes (variables) represents the sign (positive/negative) and the weight (strength) of the connection, depicting the unique associations between two nodes while controlling for all other nodes in the network (Epskamp et al., 2018). Figure 2a presents the unique associations between fundamental frequency (F0), jitter (JIT), Harmonics-to-Noise Ratio (HNR), formant 1:2 ratio (F1/2), and mean voiced segment length (VO) at rest (*n* = 148). The strongest connection in the network occurs between HNR and F0 (.75). HNR is positively associated with F0 and VO. The latter two constructs are negatively associated with one another. In addition, VO and HNR are negatively associated with JIT. For HNR, an additional negative edge emerges with F1/2. Finally, we observe a negative association between JIT and F1/2.

**Fig. 3:**
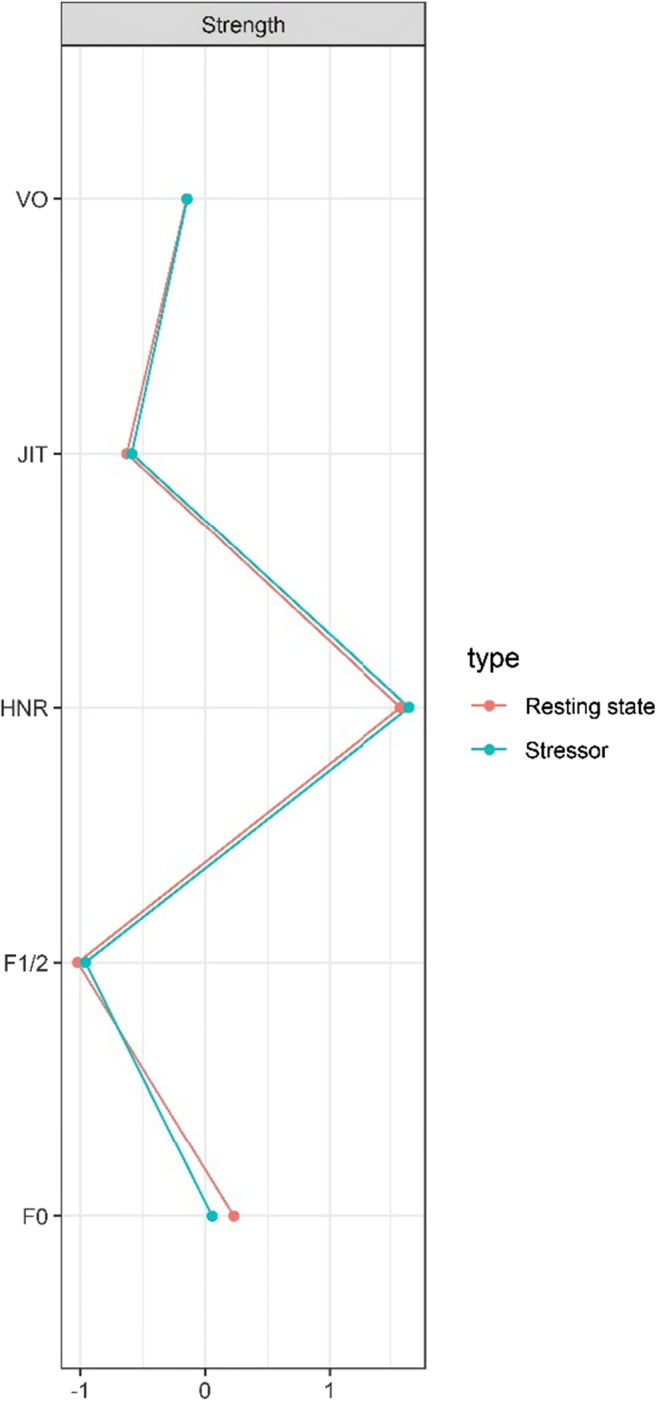
Strength centrality

The pattern of unique interrelations between the speech parameters of interest does not seem to be affected by the stress induction procedure. That is, the network model obtained based on the speech fragments that were collected immediately following the stressor (Fig. 2b, *n* = 148), is highly similar to the resting state network (Fig. 2a). This is also reflected by the indicator of node centrality (Fig. 3), which quantifies how well a node is directly connected to other nodes by adding up the strength of all connected edges to a node (Epskamp et al., 2018). In particular, in terms of node strength, HNR is the most central node in each of the networks, followed by F0, VO, and JIT. F1/2 is the least connected node in the model. This is also reflected by the amount of explained variance for each of the nodes (i.e., node predictability). In particular, node predictability of HNR was .85 and .83 in the resting state and stress network, respectively, whereas only 24% and 31% of the variance in F1/2 was explained by the neighboring nodes in the resting state and stress network respectively (see Table 1 for estimates of node predictability and supplemental material for, edge accuracy, edge differences, and centrality stability).

**Table 1:** Node predictability for pre-stressor network (aim 1), post-stressor network (aim 1), and change network (aim 2)

Node	R^2^ Pre-stressor network	R^2^ Post-stressor network	Change network
F0	.78	.72	.16
HNR	.85	.83	.37
JIT	.41	.42	.35
VO	.40	.44	.19
F1/2	.24	.31	.12
NA			.02

In line with the visual interpretation of the obtained network models, a statistical comparison of the models suggested strong overlap. That is, we observed a correlation of *r* = .99 between the adjacency matrices of both networks. Similarly, centrality strength and node predictability for the resting state and post stressor networks each reached *r* = .99. Indeed, the network comparison test suggested no significant differences in terms of overall network structure (M = 0.06, *p* = .93; network invariance test) or strength of connectivity (resting state network = 2.33; post stressor network = 2.44; *S =* 0.11, *p =* .57; global strength invariance test).

## Modeling the unique associations between stress reactivity and change in speech parameters (aim 2)

**Fig. 4:**
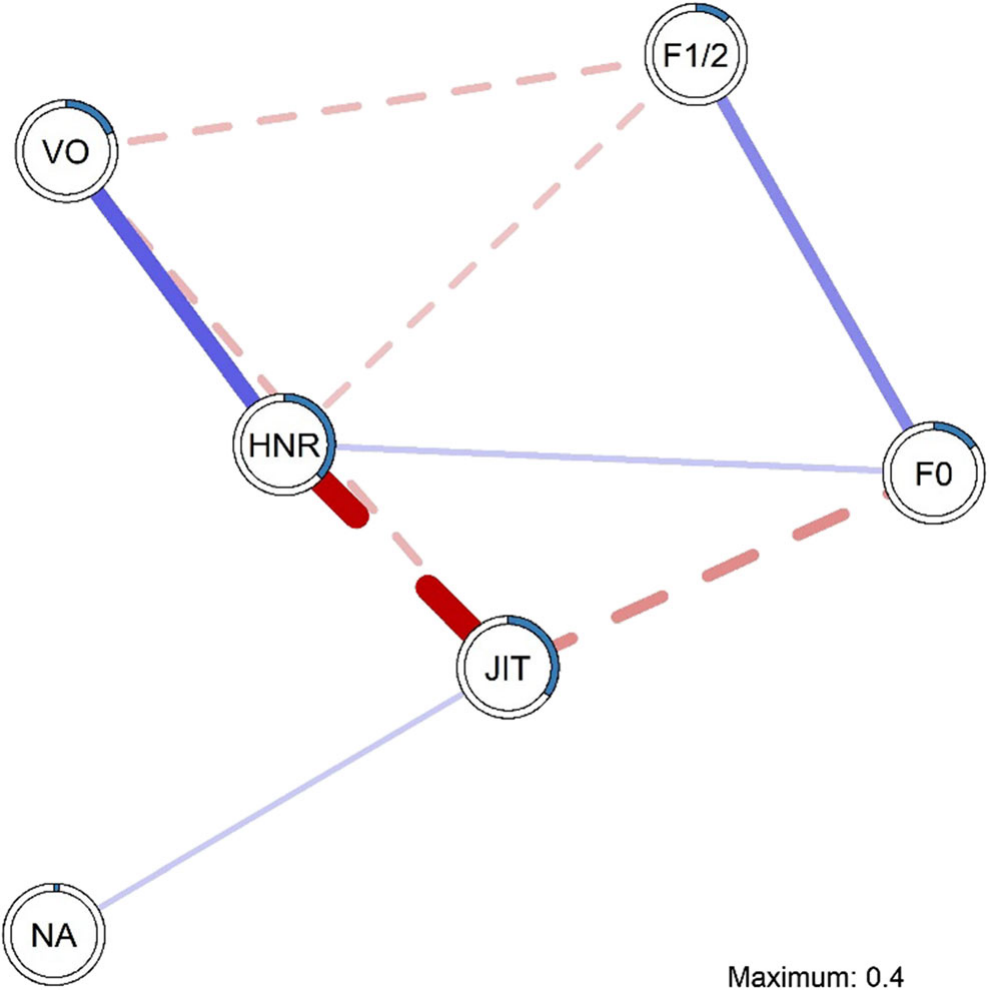
Unique associations between change in negative affect and speech parameters. *Note.* Edges in the model represent the unique associations between each of the nodes. Edge thickness reflects the strength of association, where strong associations are presented using thicker edges. *Blue/Full edges* represent positive associations, whereas *red/dashed edges* represent negative associations; the edge weights presented in the model can also be found in the edge weight matrix (Supplemental table 8). Node predictability (R^2^) is visualized as a pie chart around each node and can also be found in Table 1

Figure 4 (*n* = 148) depicts the unique associations between change in negative affect (NA) and change in the speech parameters following the stress induction procedure with every edge (connection) between two nodes (variables) represents the sign (positive/negative) and the weight (strength) of the connection, depicting the unique associations between the two nodes while controlling for all other nodes in the network (Epskamp et al., 2018). Interestingly, change in JIT was the only speech parameter that was directly connected to change in NA. In particular, the experience of more negative affect throughout the stress induction procedure was directly related to increased JIT. All other speech parameters were only indirectly connected to change in NA through JIT. Change in JIT was negatively related to change in HNR and F0, and VO, which suggests that increases in JIT due to the stress induction procedure were related to decreases in HNR, F0, and VO. In addition, we observed positive associations between HNR and VO/F0, and F0 and F1/2. Finally, we observed negative associations between F1/2 and VO/HNR. Based on node strength, change in HNR and jitter emerged as the most central nodes in the network, whereas change in NA was the least central node (see supplemental material for estimates of node predictability, edge accuracy, edge differences, and centrality stability).

## Discussion

The two aims of the present study were to gain insight into (1) the unique associations between specific speech parameters (fundamental frequency, jitter, Harmonics-to-Noise Ratio, voiced segment length, and formant 1:2 ratio) after as compared to before experiencing psychosocial stress, and (2) how change in these speech parameters was uniquely associated with change in self-reported NA following the stressor. We measured (change in) speech in the context of experimentally induced stress in a large sample of individuals selected from the community. The psychosocial stress induction was successful, as evidenced by higher skin conductance levels after the stress induction as compared to baseline, as well as increased negative affect following the stress induction. Moreover, we observed a significant positive association between these measures, indicating that the more skin conductance levels were increased following the psychosocial stress induction, the more negative mood was reported, providing support for the validity of the stress-induction method. As such, the comparison between the resting state and stress network allows us to test for changes in interrelations between the speech parameters after experiencing stress (aim 1).

First, network analyses were conducted on the selected speech parameters of interest at baseline, representing the unique associations between each of these parameters in a resting non-stressed state. This network shows Harmonics-to-Noise Ratio (HNR) as the most central node, being connected to all other speech parameters. The strongest connection that occurs is the positive connection between HNR and fundamental frequency (F0), implying that less noise is present in higher-pitched voices and vice versa, as has been reported by Ferrand (2002). Furthermore, results show that several connections between most parameters are observed, which differ in their strength and orientation, indicating an interacting and cohesive network.

When comparing this baseline network with the post-stressor network, no differences between the interrelations of the different speech parameters were observed, suggesting that the relations between the parameters (selected as nodes in the current study) do not change after a stress induction procedure. More specifically, in both models, (1) HNR emerged as the most central node, (2) the strongest connection was observed between HNR and F0, and (3) all parameters were connected to at least two out of four other nodes in the network. The fact that the network of speech parameters was highly similar after versus prior to the stress induction procedure is an interesting and innovative observation, as it shows that the complex interplay between each of the above presented core speech parameters is not impacted by stress, and as such cannot be used as an indicator for stress. In particular, the unique interrelations remained stable in a stressed versus a non-stressed state.

In addition to comparing the pre- and post-stress networks of speech parameters, we composed an individual network model of change (delta) scores of each of the parameters and self-reported negative affect to gain insight into the unique relations between speech parameters and individual differences in stress reactivity (aim 2). We found that changes in jitter (JIT), a fairly central speech parameter in the estimated network, were directly positively related to changes in self-reported negative affect, after controlling for the influence of other parameters in the network. Even though after the regularization procedure the strength of this association was relatively weak, this finding is important as it suggests a unique association between speech and self-reported negative affect. Jitter quantifies the modulation of the periodicity of the voice signal and as such is related to the amplitude variation of the sound wave and is mainly affected by the lack of control of vibration of the cords (Teixeira et al., 2013). Increased jitter has been observed in pathological voices (Teixeira et al., 2013) and in physical task stress (Koblick, 2004), whereas decreased jitter is often discussed in the context of psychological stress (Giddens et al., 2013; Van Puyvelde et al., 2018). Yet, the literature is inconsistent, as both a decrease and an increase in jitter have been observed with increased stress in different task designs (Giddens et al., 2013). However, jitter has not been reported in relation to negative affect (Giddens et al., 2013). In early studies, it has been suggested that jitter decreases in direct relation to stress levels (as described in Giddens et al., 2013; Van Puyvelde et al., 2018) and pointed out that jitter might be a better indicator of stress than F0 (Hecker et al., 1968; Mendoza & Carballo, 1998). More recent studies have shown jitter to be a crucial feature in the classification of stress and emotion (e.g., Li et al., 2007; Rothkrantz et al., 2004). However, jitter has especially been highlighted in the field of speech pathology, being mainly affected by a lack of control over the vibration of the cords, which could explain its occurrence in psychosocial stress (Teixeira et al., 2013). As such, the unique association between the change in jitter and the change in self-reported negative affect following a potent psychosocial stressor, while controlling for other effects and variables, opens a new avenue to the research field of speech parameters in the context of psychological stress.

Interestingly, even though the network model of the current study depicts a direct connection between change in self-reported negative affect and change in jitter, jitter is by itself strongly linked to several other speech parameters in the network model. A direct connection with F0 was to be expected considering that jitter represents the variations that occur in the fundamental frequency (F0). Moreover, especially a strong association between change in jitter and change in HNR is observed, which together with jitter form the most central nodes of the network. Prior studies have demonstrated that HNR is more sensitive to subtle differences in vocal function than is jitter (Awan & Frenkel, 1994). Although direct connections between the other speech parameters and negative affect were expected, such as a positive unique association between negative affect and F0 (Giddens et al., 2013), our findings suggest that these parameters function through jitter in their connections to changing mood in the context of psychosocial stress. It could be argued that, at least to some extent, these parameters function through HNR and its strong interplay with jitter too.

To the best of our knowledge, this study is the first to examine the impact of psychosocial stress on the unique interrelations between key speech features, and how change in these parameters in the context of psychosocial stress relates to change in self-report measures for stress (i.e., negative affect). Our findings provide several implications for the measurement of speech in the context of psychosocial stress, as well as for the measurement of stress via speech features. That is, our findings point towards the stability of the network structure of speech features in the context of stress, and the role of jitter as the only speech feature which showed a direct association with self-reported negative affect, suggesting the importance of jitter in the context of stress assessment via speech. The present study used a standardized method of psychosocial stress induction in a highly controlled lab setting. The analysis has been conducted using an exploratory and data-driven method which allows to model complex interrelations in an intuitive manner. Therefore, the present study’s main strength is the generation of trustworthy hypotheses.

Future studies using large sample sizes whilst maintaining a within-subject design in a controlled setting are absolutely warranted. On the other hand, considering the accessibility of high-quality microphones, combining frequent speech recordings with continuous smartwatch recordings of heart rate and skin conductance will generate more dynamic results that could withstand and overcome prior limitations of controlled lab settings and can uncover the stability and strength of the different relations. However, this is to be confirmed by basic experimental research investigating the complex relation between speech and stress in a well-controlled setting, which was the aim of the current study. Finding the key parameters of stressed speech and being able to use these to assess stress levels in a wide variety of settings, swiftly and cost-effectively, will enable us to monitor excessive stress levels and set up interventions where necessary.

Even though the current study has several strengths such as such as its innovative nature and ecological validity, some limitations should be discussed. Firstly, it should be noted that network models are merely descriptive rather than predictive. These networks are undirected and therefore do not allow any statements regarding the direction of the observed effects. This data-driven, explorative analysis, is hypothesis generating as the identified paths in the networks might be indicative of causal relationships which should be tested in future prospective or experimental research. Secondly, as network models value each individual relation between the different parameters in an unguided manner, we were limited in the number of parameters that could be included in the model due to the sample size. The current set of parameters was selected based on literature research and has brought forth a network of interesting relations. However, an expanded network would give more insight into the stability of these relations, as well as further explain the dynamics between speech parameters and negative affect. Thirdly, the required larger sample size to do so would also increase the strength of the network comparison (resting state vs. stressed) made. However, in this context both networks were highly similar. As such, the non-significant findings for the network comparison test are unlikely to be driven by a lack of power. Overall, jitter seems to be a central node in the relation between speech and negative affect, which should therefore be further studied using confirmatory analyses. Fourthly, due to some technical setbacks, most of the collected data for ECG and EDA was not usable. This is especially unfortunate as this would give insight not only into the interplay between speech and self-reported negative affect but also into the relations with other indicators of objectively experienced stress (e.g., biomarkers).

### Conclusions

Stress has long been a much-discussed topic, and as such many different methods for stress measurement have been proposed over the years. Recently, speech analysis has been proposed as a possible physiological marker for stress which can be measured in a remote and non-invasive matter. The current study deployed network analysis to investigate the unique associations between specific speech parameters prior to and following exposure to a psychosocial stressor (aim 1), and to model the unique associations between specific speech features and self-reported stress (i.e., experienced negative affect; aim 2). For this purpose, we relied on a well-validated stress induction procedure in a controlled lab setting. The network of speech parameters was highly similar after versus before the stress induction, suggesting that the complex interplay between each of the used speech parameters was not impacted by stress. Interestingly, changes in jitter were directly positively related to changes in self-reported negative affect, indicating that this speech feature may be of particular interest in the context of stress assessment. These findings warrant further investigation in the diagnostic value of speech features to monitor stress in daily life, which requires intensive time series data.

## Data Availability

The processed datasets generated during and/or analyzed during the current study are available openly online through https://osf.io/7byzh/ The complete raw dataset is available upon reasonable request rather than openly accessible online due to the sensitivity of speech recordings with regards to participants’ identities.
